# Burden of early, advanced and metastatic breast cancer in The Netherlands

**DOI:** 10.1186/s12885-018-4158-3

**Published:** 2018-03-07

**Authors:** G. T. Vondeling, G. L. Menezes, E. P. Dvortsin, F. G. A. Jansman, I. R. Konings, M. J. Postma, M. H. Rozenbaum

**Affiliations:** 1 Department of Pharmaco-Epidemiology and Economics, University of Groningen, PO Box 72, Groningen, 9700 AB The Netherlands; 2 Department of Public Affairs, Pfizer, Rotterdam, The Netherlands; 3 Department of Radiology and Nuclear Medicine, University Medical Center Utrecht, Utrecht, The Netherlands; 4 Department of Clinical Pharmacy, Deventer Hospital, Deventer, The Netherlands; 5 Groningen Research Institute of Pharmacy, PharmacoTherapy, - Epidemiology & -Economics University of Groningen, Groningen, The Netherlands; 6 Department of Medical Oncology, VU University Medical Center, Amsterdam, The Netherlands

**Keywords:** Breast cancer burden costs incidence prevalence mortality early advanced metastatic

## Abstract

**Background:**

The aim of this study was to estimate the total economic and health related burden of breast cancer in the Netherlands.

**Methods:**

Data on incidence, prevalence, mortality and survival were extracted from the Dutch National Cancer Registry and were used to calculate the economic and health related burden of breast cancer for overall, DCIS (stage 0), early- (stage I), locally advanced- (stage II-III) and metastatic- (stage IV) breast cancer by age groups and by year (if applicable).

**Results:**

The overall incidence of breast cancer increased from 103.4 up to 153.2 per 100,000 women between 1990 and 2014. The increase was driven by DCIS and early breast cancer as the incidence of locally advanced and metastatic breast cancer remained stable. Between 1990 and 2014, ten-year overall survival rates increased from 87% to 93% for early breast cancer, 41% to 62% for locally advanced- and from 6% to 9% for metastatic disease. Annually, breast cancer in the Netherlands is responsible for approximately 3100 deaths, 26,000 life years lost, 65,000 Disability Adjusted Life Years (DALYs) and an economic burden of €1.27 billion.

**Conclusions:**

This study provides a comprehensive assessment of the burden of breast cancer and subsequent trends over time in the Netherlands.

**Electronic supplementary material:**

The online version of this article (10.1186/s12885-018-4158-3) contains supplementary material, which is available to authorized users.

## Background

Breast cancer is the most common cancer and leading cause of cancer-related mortality amongst women worldwide [1, 2] .Together with lung, colorectal and prostate cancer, breast cancer contributes to half of the overall burden of cancer mortality in Europe [3] .In Europe approximately 500,000 women are diagnosed with breast cancer annually and in 2012, breast cancer cases were responsible for a third of all cancer related deaths (131,259) [4]. The incidence and prevalence of breast cancer increased over the past decades due to the progressive nature of the disease and the aging population in Western countries [1] .In 2010, the lifetime risk of developing breast cancer was 1 in 6.6 women in the Netherlands while the lifetime risk of breast cancer death was 1 in 27 [5] .Furthermore, it has been shown that the Netherlands has a breast cancer related Disability Adjusted Life-Year (DALYs) burden of approximately of 1100 DALYs per 100,000 women [6].

Several previous studies aimed to assess the burden of breast cancer. However, to our knowledge, there is currently no study available assessing both the economic and health Burden of Disease (BoD) stratified by stage using data from a national cancer registry over a 24 year period [7, 8] .Stratification by stage of diagnosis is necessary, since the burden of disease differs between disease stages [7] .Mapping the stage stratified breast cancer burden over time also enables adequate evaluation of policy implementation and decision making, for instance regarding the mammographic screening program [9] .In addition, this will provide new insights into the difference in morbidity and mortality between early, locally advanced and metastatic breast cancer.

The aim of this study is to describe the total health and economic related burden of breast cancer in the Netherlands. The total burden of breast cancer is expressed in economic costs per year, incidence, prevalence, survival, mortality, life-years lost and DALYs. The results of this study can be used to facilitate healthcare decision making, resource allocation and investment studies.

## Methods

### Data source

Breast cancer incidence, prevalence, survival and mortality data between 1990 and 2014 were obtained from the Netherlands Comprehensive Cancer Organization (IKNL) and the National Cancer Registry (NCR) [10] .As survival data within this database is generated in cohorts, most recent 10-year survival was available until 2009. Additionally, data was available for up to 4-year survival in the latest cohort (2010–2013). Tumors in this registry are coded according to the International Classification of Diseases for Oncology (ICD-O). Regarding multiple primary cancers, the registry follows the guidelines developed by the International Agency for Research on Cancer (IARC) and the International Association of Cancer Registries (IACR). In this study we differentiate between early, locally advanced and metastatic breast cancer based on the Tumour Node Metastasis (TNM) classification and IACR guidelines [11, 12] .Stage 0 (DCIS) and Stage I were defined as early breast cancer, stages II to III as locally advanced breast cancer, and stage IV was labeled as metastatic breast cancer. In the performed analyses (see below), the results are reported per separate stage where possible. This distinction was made based on the differences in treatment guidelines with recommendations ranging from breast-conserving surgery to mastectomy, radiation therapy, chemotherapy, hormone therapy, or a combination of different treatments in stage IV [13, 14].

### Incidence, prevalence, and mortality

Incidence, prevalence and mortality data were both obtained in crude rates (absolute number of cases in the Netherlands), and in European Age Standardized Rates (ESR) (cases per 100,000 women, corrected by age for the European geographic composition). DCIS is not included in the survival analysis. Up to 10-year overall survival data (over the period 1989–2009) were available. For the most recent data a sub-analysis consisting of 4-year survival data from the period 2010–2013 was included. When applicable, results are presented in five-year age groups starting at the age of 15 (e.g. 15–19, 20–24, etc.) up to 95, after which the age band is open including all subject aged 95 years and older.

### DALY burden

The Disability Adjusted Life-Year (DALY) is a measure to express the BoD. DALYs represent the number of healthy life lost due to a disease or risk factor. One DALY can be regarded as 1 lost year of “healthy life”. It is calculated by adding the years of life lost due to premature mortality (YLL) to the years lived with disability (YLD, morbidity). YLL regards the sum of years that a person would have lived if the individual would not have the current disease under study and was based on the average life expectancy of women in the Netherlands [15] .YLD expresses the consequences of living with a less than perfect health condition, and is estimated based on the length of time with that condition and the corresponding disability weight. The parameters used to estimate DALYs are mortality, incidence, average duration to death or cure from breast cancer, the disability weight of living with breast cancer and the age at diagnosis. Based on the EUROCARE-4 study, we estimated that the median duration of cancer until patients were either cured or deceased was 4.3 years [16] .This number was subsequently used to calculate the YLL. The Disability weight was assessed at 0.38, that was derived from Disability Weights for Diseases in the Netherlands study, being the average disability weight of all disease stages, weighted by its duration and cure rate [17] .A discount rate of 1.5% was applied to both YLL and YLD, which is the standard discount rate in the Netherlands for discounting health outcomes and utilities. Additionally, the DALY burden was calculated without inclusion of DCIS. The metrics on how DALYs were calculated can be found in Additional file 1.

### Economic burden

The total healthcare expenditure due to breast cancer in the Netherlands was obtained from the cost of illness database by the national institute for public health (RIVM). Data were available for 2003, 2005, 2007 and 2011. This database is a product of the Dutch government and was used for policy and healthcare decision making. The costs include public healthcare and prevention, costs for 1st line care, hospital & medical professional care, elderly care, drugs, management and costs of other healthcare providers, as derived from the national cost of illness database [18] .The costs of breast cancer in 2014 were estimated by extrapolating those from 2011 using Dutch Customer Price Index (CPI) inflation rates [19] .Productivity losses due breast cancer-related morbidity and mortality in the Netherlands were estimated using the human capital approach (more details on the exact methods and equations used to calculate these indirect non-medical costs can be found in Additional file 1). All prices in this study are expressed in 2014 Euros.

### Sensitivity analysis

Univariate sensitivity analysis was used to investigate the sensitivity of the base-case estimates to variation in several parameters. The gross domestic product growth rate, used for the prediction of future growth rate, was varied to 0%, 1.5% and 3.5%, to account for uncertainty over future growth in the Dutch economy. The discount rate for costs was varied between 2% and 6%. Female Gross Labor participation was adjusted to match the amount of men, to account for future emancipation. Work-Absence rates were varied by ±10% to investigate the sensitivity of this estimate on productivity losses due to morbidity. In addition, the effect of extending the retirement age from 65 to 66 and 67 was explored, to account for potential changes in the official retirement age in the Netherlands. For productivity losses due to mortality, we varied the results by using the friction cost method, which only takes the friction period of 85 calendar days in consideration for potential loss of productivity [20].

## Results

### Absolute cases, incidence, and prevalence

In total 320,179 women were diagnosed with breast cancer in the Netherlands between 1990 and 2014. The absolute overall number of cases per year increased from 8233 in 1990 up to 16,688 in 2014 (see Table 1 and Appendix Figure 1). This increase was mainly due to the 231% increase in cases of early breast cancer (including DCIS) from 2682 to 8869. During the same period, the number of locally advanced breast cancer cases also increased by 40% and metastatic disease increased by 43%. When excluding DCIS from the analysis the absolute overall number of incident breast cancer cases increased from 7913 in 1990 to 14,541 in 2014.

**Table 1:** European Standardized Incidence Rates of breast cancer per stage of diagnosis for the Netherlands between 1990 and 2014, along with the absolute number of cases between brackets

	Early Breast Cancer			Advanced Breast Cancer				Metastatic	Overall
Period	DCIS	I	Total	II	IIIa	IIIb/c	Total	IV	DCIS (0) - IV
1990	4.2 (320)	30.4 (2362)	34.6(2682)	51.7 (4151)	3.9 (301)	6.5 (556)	61.1(5008)	6.7 (543)	103.4 (8233)
1995	10.0 (793)	43.4 (3501)	53.4(4294)	51.9 (4425)	3.5 (294)	5.3 (534)	60.7(5253)	5.4 (493)	119.5 (10040)
2000	12.2 (1059)	48.1 (4262)	60.3(5321)	58.5 (5355)	3.6 (324)	4.9 (536)	67(6215)	5.6 (562)	132.9 (12098)
2005	12.3 (1229)	50.4 (4738)	63.7 (5967)	49.7 (4937)	9.5 (903)	6.9 (752)	66.1 (6592)	5.7 (612)	135.5 (13171)
2010	19.3 (1877)	60.3 (6083)	79.6 (7960)	45.8 (4884)	9.0 (895)	7.6 (844)	62.4 (6623)	5.1 (573)	147.1 (15156)
2014	20.4 (2127)	62.3 (6742)	82.7 (8869)	49.1 (5431)	8.4 (867)	6.2 (726)	63.7 (7024)	6.8 (775)	153.2 (16668)
Percentage increase	+ 386% (+ 565%)	+ 105% (+ 185%)	+ 139% (+ 231%)	−5% (+ 31%)	+ 115% (+ 188%)	−5% (+ 31%)	+ 3% (+ 40%)	+ 1% (+ 42.7%)	+ 102% (+ 103%)

**Fig. 1:**
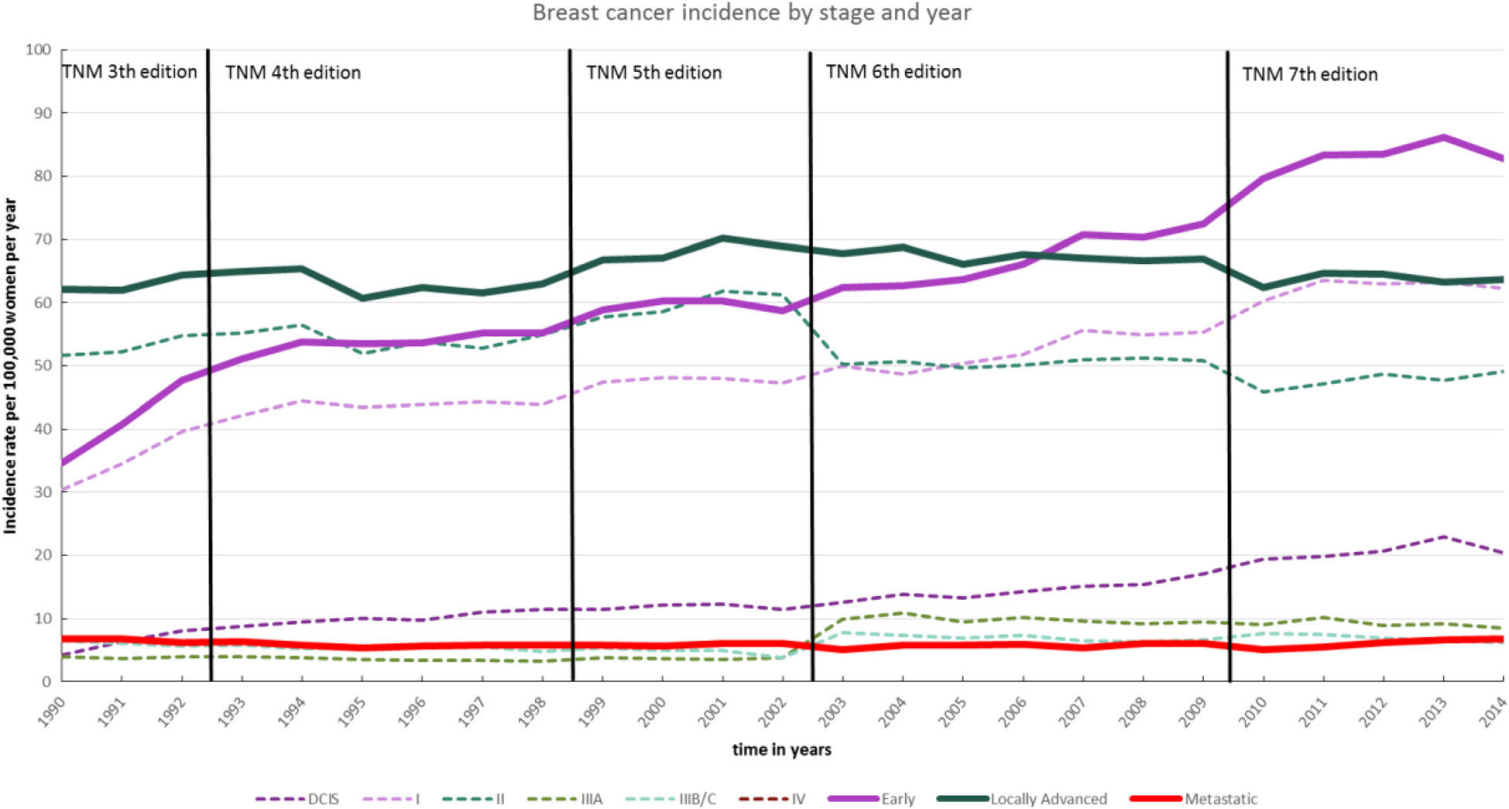
European Standardized breast cancer incidence rates per 100,000 women stratified by stage of diagnosis between 1990 and 2014 for the Netherlands. Vertical lines represent changes in TNM

The overall incidence increased from 103/100,000 in 1990 to over 153/100,000 women in 2014 (Table 1 and Fig. 1). The increase in the incidence was mainly attributable to an increase of early breast cancer diagnosis (from 34.6/100,000 to 82.7/100,000 women). In particular the incidence of DCIS increased by 386% (from 4.2/100,000 to 20.4/100,000). Overall the incidence of locally advanced breast cancer was stable (3% increase). However, within more advanced disease stages, there were large differences with a decline of 5% for both stage II and stage IIIb/c and an increase of 115% in stage IIIa. Finally, the incidence of metastatic breast cancer (stage IV) remained stable during the study period.

The 5-year prevalence of breast cancer increased by 77% (from 41,476 in 1995 towards 73,261 in 2014). The 10-year prevalence (available from 2000 onwards) increased by 58% (from 78,892 to 124,996).

### Proportional distribution by stage over time and by age

**Fig. 2:**
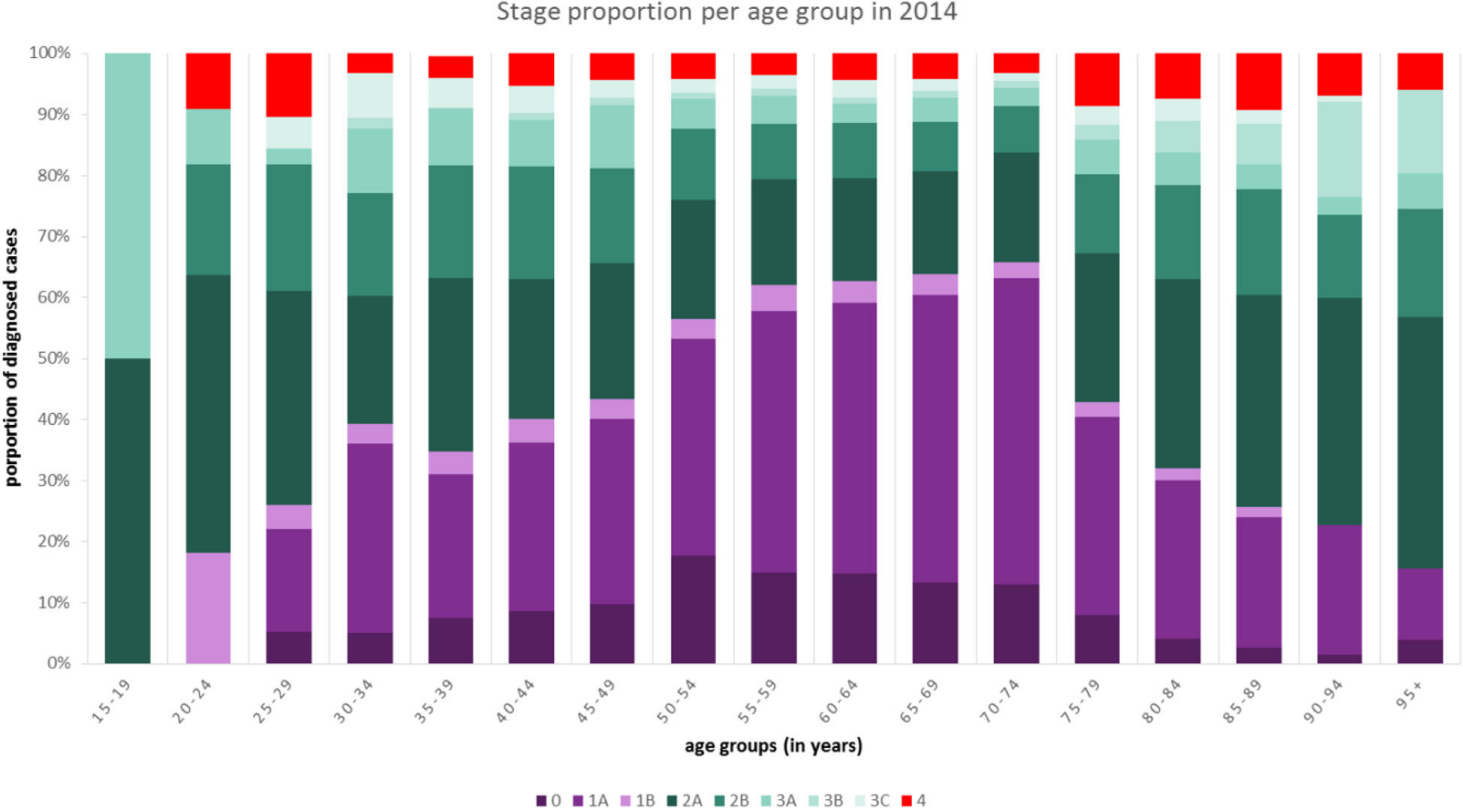
Proportionate stage at diagnosis per age group for the year 2014

**Fig. 3:**
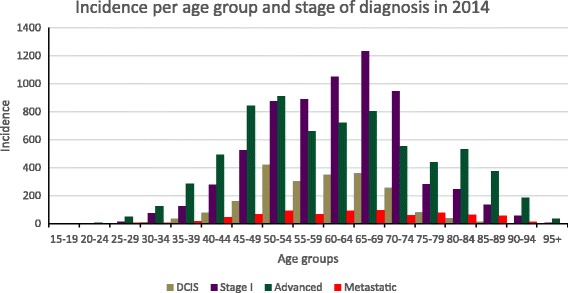
Incidence of early and advanced breast cancer by age for 2014

The proportion of women diagnosed with early breast cancer (including DCIS) increased from 33% in 1990 to 54% in 2014. During the same period, we observed a reduction in the proportion of locally advanced breast cancer (from 60% to 42%) and metastatic disease (from 6% to 4%). Additional analysis showed that the proportion early breast cancer increased from 31% in 1990 to 47% in 2014 while locally advanced and metastatic decreased from 63% and 7% to 48% and 5% respectively when DCIS was excluded. Figure 2, shows the proportional distribution by stage at diagnosis per age group for 2014. It clearly shows that the proportion of early stages of breast cancer is highest in the group of women aged between 50 and 74, which corresponds to the breast cancer screening-bracket in the Netherlands (50 to 75 years). Also the incidence (in absolute annual numbers) is higher in the age groups within the screening brackets **(**Fig. 3**).** This is also true for locally advanced and metastatic incidence. The exception is that the number of cases of locally advanced disease is higher in the 45 to 49 year old group compared to the age groups within the 55 to 75 years range.

### Survival rates

**Fig. 4:**
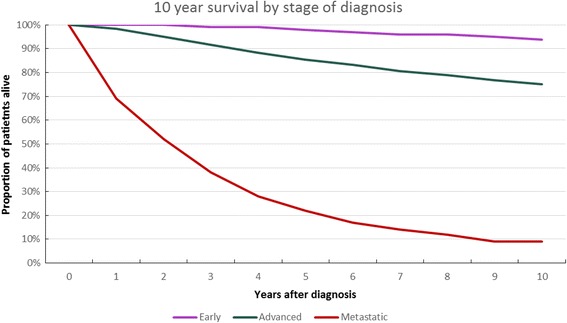
Ten-year overall survival of early and advanced breast cancer patients

Figure 4 shows the overall survival for early, locally advanced and metastatic breast cancer between 2003 and 2009 (6th TNM edition). It clearly shows the large impact of stage at diagnosis. For example, 5 years after diagnosis the overall survival was 98% for early breast cancer, 85% for locally advanced and 22% for metastatic disease. After 10 years, these numbers are 94%, 75% and 9%. Survival data specified per cancer stage from Ia to stage IV is available in the Additional file 1.

An increase in survival was observed for all stages of breast cancer compared to historical survival data (see Additional file 1: Tables S1-S5). Accordingly, the most recent data in the 7th TNM edition show a slight increase in survival. Although our data analysis regarded a limited period of four-years (2010–2014), we observe an increase in overall survival of 1% for early breast cancer, 1% locally advanced and 4% metastatic breast cancer in comparison with the previous survival data from the 6th TNM edition.

### Deaths, life years and DALYs lost

**Fig. 5:**
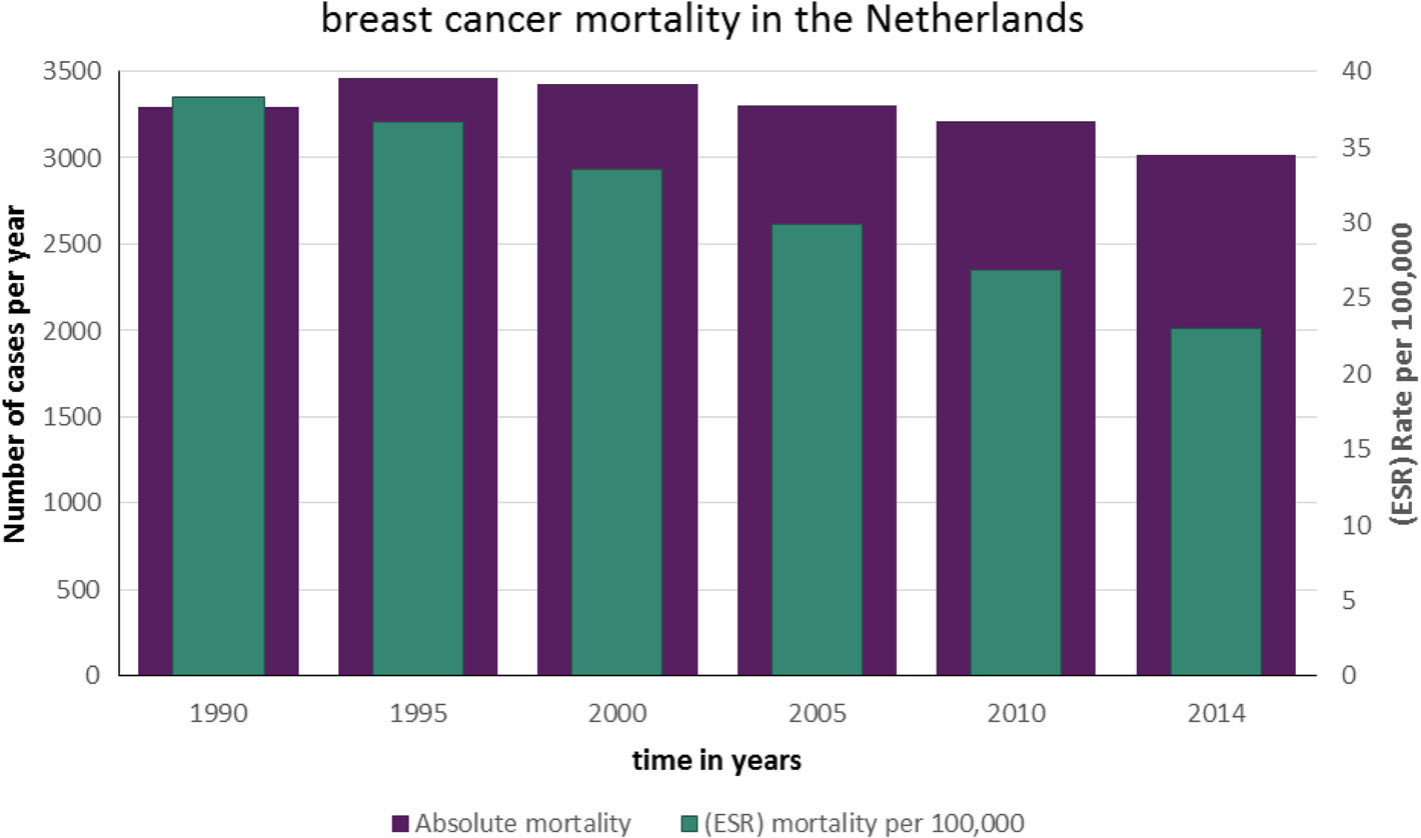
Mortality in the Netherlands between 1990 and 2014

In total 84,282 women died of breast cancer in the Netherlands between 1990 and 2014. The number of deaths of 3666 was highest in 1999 and decreased to 3014, i.e. by 18%, in 2014 (see Fig. 5). Corrected for age and for the demographic composition of the European population, a more significant decrease of 41% was observed (from 39.17 per 100,000 women per year in 1991 to 23.01 per 100,000 in 2014). The total number of life-years lost due to breast cancer between 1990 and 2014 in the Netherlands was estimated at 1.35 million. Despite the rising incidence in breast cancer, the total number of life-years lost per year decreased by 15.6% during this same period (Additional file 1: Table S6).

**Fig. 6:**
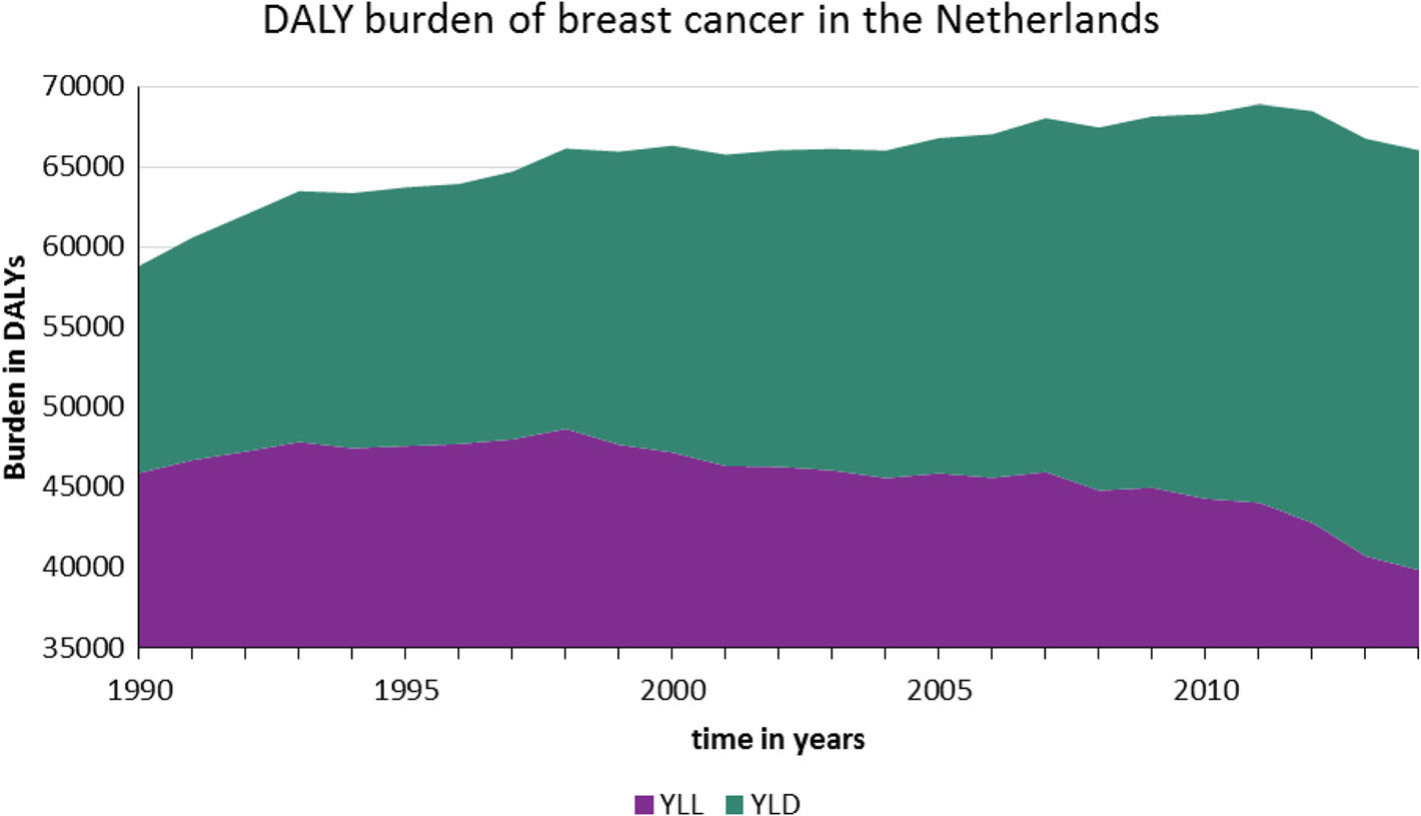
Burden of breast cancer in the Netherlands expressed in Disability Adjusted Life years (DALY) which consist of life-years lost due to early mortality (YLL) and life-years lost due to morbidity (YLD)

The DALY burden peaked in 2010 when breast cancer was accountable for a total of 68,500 DALYs in the Netherlands (Fig. 6). From this total, 50,000 where attributable to life years lost from early mortality (YLL) and 18,500 from disability (YLD). Since 1990, the YLL gradually decreased from 45,900 to 38,600, while the YLD increased from 13,000 to 26,000 which outweighs the reduction in YLL. Excluding DCIS from the analysis resulted in a decrease in the YLD leading to a total burden of 61,600 DALYs in 2014 versus 65,000 with inclusion of DCIS. In terms of the age distribution, patients aged 45–65 are accountable for the highest number of DALYs, which is directly correlated with the incidence and mortality of the disease.

### Healthcare costs and productivity losses

**Fig. 7:**
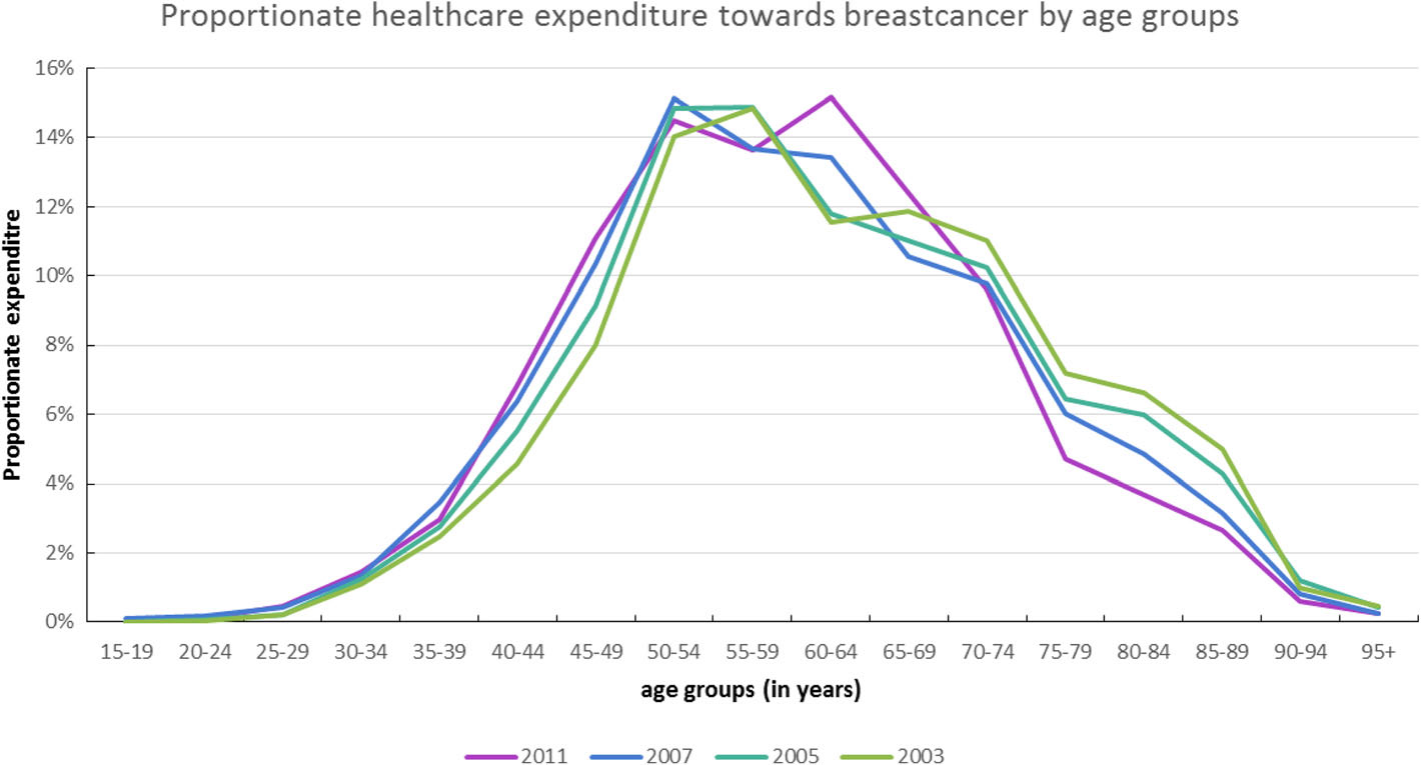
Proportionate healthcare spending of breast cancer by age groups in the Netherlands

The total healthcare costs of breast cancer in the Netherlands increased from €199 million in 2003 up to €692 million in 2011 (Table 2). Costs in most categories increased in absolute terms, although regarding the proportional expenditure per year, we see that only the costs related to hospital and medical professional care increased. The costs due to hospital and medical professional care accounted for €96.5 million (49% of the total budget) in 2003 and were €542.6 million (78% of the total budget) in 2011. Proportionate drug expenditure was stable between 2003 and 2007 (13%), but dropped to 7% between 2007 and 2011. The healthcare expenditure per age group (Fig. 7) is highly correlated to the incidence per age groups (Fig. 3**).** Total cost of productivity losses due to morbidity and mortality cumulated to €260 million and €243 million annually, respectively (Table 3**)**. Combining the productivity losses with the total estimated healthcare expenditure, the total economic due to burden of breast cancer cumulates to €1.27 billion for 2014.

**Table 2:** Healthcare costs of breast cancer in the Netherlands in Million Euro’s

Expenditure	2003	2005	2007	2011	Estimates 2014
Public healthcare and prevention	41.8 (21%)	44 (18%)	47.7 (11%)	63.1 (9%)	9%
1st line care	4.6 (2%)	5.1 (2%)	7.1 (2%)	7.5 (1%)	1%
Hospital & medical professional care	96.5 (49%)	129.8 (54%)	280.7 (65%)	542.6 (78%)	78%
Elderly Care	19.3 (10%)	18.5 (8%)	19.1 (4%)	17.8 (3%)	3%
Drugs	26.7 (13%)	32.6 (13%)	57.1 (13%)	45.7 (7%)	7%
Other healthcare providers	3.1 (2%)	3.3 (1%)	5.7 (1%)	2.7 (0%)	0%
Management	6.7 (3%)	8.9 (4%)	12.2 (3%)	12.5 (2%)	2%
Total Costs in Million (€)	199 (0.31%^a^)	243 (0.36%^a^)	430 (0.58%^a^)	696 (0.78%^a^)	768 (0.80%^a^)

^a^Percentage of healthcare budget

**Table 3:** The total economic burden caused by breast cancer in the Netherlands (Human Capital Approach)

Healthcare Expenditure towards breast cancer in 2014	€768 million
Productivity Losses due to morbidity	€260 million
Productivity Losses due to mortality	€243 million
Total economic burden	€1271 million

### Sensitivity/scenario analyses

Additional file 1 summarizes the results of the sensitivity analysis for the calculation of productivity losses due to morbidity and early mortality. It shows that the estimated productivity losses due to morbidity were between €258 and €334.6 million annually. Productivity losses due to mortality ranged between €221 million and €323 million annually. A scenario analysis that took future emancipation into account by matching the female gross labor participation to that of men, resulted in a total productivity of €335 million due to morbidity and €317 due to early mortality. Applying the friction cost rather than the human capital approach resulted in a productivity loss of €43 million due to morbidity and €6 million due to early mortality.

## Discussion

This is the first study in The Netherlands to examine the burden of breast cancer in terms of incidence, prevalence, survival, deaths, life years lost, DALYs, healthcare and productivity losses stratified by breast cancer stage and age using data from a national registry. This study shows an overall increase in incidence of breast cancer between 1990 and 2014 which was mainly attributable due to an increase in the incidence of early breast cancer. While the incidence increased the mortality rates decreased for both early, locally advanced and metastatic breast cancer during the same time period. Despite the promising trends in survival breast cancer remains the leading cause of cancer related deaths amongst women. The rising incidence corresponds with an increase in the total economic burden of breast cancer, for which the majority of the costs concern healthcare costs followed costs for productivity losses due to morbidity and early mortality.

Although DCIS (stage 0) may not be considered as breast cancer in every country, it still needs therapeutic intervention (and in some countries hormonal treatment), has an impact on the quality of life of the patients and is associated with (treatment) costs. Therefore DCIS was included in the analysis. During the study period the incidence of early stage breast cancer increased while the incidence of locally advanced and metastatic breast cancer remained stable. The biggest rise in incidence was seen within DCIS followed by stage I diagnosis. This discrepancy could partly be explained by the introduction and continuous improvement of the national mammographic screening program, resulting in an increasing number of women diagnosed with early stage breast cancer [21, 22] .These early breast cancer cases might, however, remain asymptomatic throughout a woman’s life. If such a patient undergoes unnecessary treatment, with its associated morbidity, this is considered as overdiagnosis [21, 22] .In the literature, estimates on breast cancer cases from overdiagnosis range between 0% and 36% of the total diagnosed cases [21, 22] .In contrary to the rising incidence the breast cancer related mortality decreased between 1990 and 2014. Although the available 10-year survival data was limited to 2009, this data is still relevant as is shows ongoing trends regarding decreasing mortality. This decrease can be explained by the improved diagnostics, better treatment options including radiotherapy and targeted therapies, and the introduction of more effective drugs during this period [23, 24] .The increase in economic burden was mainly caused by a rise in costs related to hospital and medical professional care. Proportionate drug expenditure was small and stable at 13% between 2003 and 2007, and dropped further down to 7% of the total expenditure towards breast cancer between 2007 and 2011. Although data on healthcare expenditure and costs for breast cancer were limited to 2011, this data is still relevant as it shows trends over time regarding increasing healthcare costs that are still ongoing and relevant for nowadays situation. Since the healthcare costs have increased in general over the last 6 years, our cost estimates might be conservative. The increase seen in indirect non-medical costs was attributable to and corresponding with the increase in incidence. The productivity losses due to morbidity where main driver of the increasing burden over time, even when DCIS was excluded from the analysis.

### Comparison to other studies

Similar to our findings, other studies also showed that incidence of breast cancer is increasing. Between 1990 and 2013, age standardized incidence rates of invasive breast cancer increased by 17% globally (44 to 52 per 100,000) [8] .However, a large difference between developing countries (a 48% increase from 28 to 40 per 100,000) and developed countries (a 8% increase from 70 to 75 per 100,000) was observed [8, 25] .Our results show an increase of 34% (from 99 to 133 cases per 100,000) during the same period. Prior studies on recent trends in Belgium, Norway and Spain sho4wed a similar increase in incidence from the 1990s and onwards [26–28] .Belgium has the highest incidence in Europe with 147.5 per 100,000 women in 2012, compared with 131.3 per 100,000 in the Netherlands. Together with Germany (122.0), the UK (129.2), Denmark (142.8) and Finland (121.0) these countries are leading in Europe [3] .Southern European countries, such as France, Spain, Portugal and Italy along with Norway and Sweden fell in the incidence range between 78.2 and 119.6 per 100,000 women and the lowest incidence was seen in eastern Europe were incidence rates were below 78.1 per 100,000 [3].

Next to the extensive screening program, the more significant increase in the incidence in the Netherlands compared to other developed countries might be explained by risk factors as late motherhood and low number of off-spring in the Netherlands as compared to other countries [29–33].

Breast cancer survival rates vary greatly between European countries, which could be attributable to the differences in stage of diagnosis in various countries. The Netherlands is leading in terms of survival when comparing out data to other countries in Europe [34] .For example, the one-year survival for stage IV disease between 2003 and 2009 was 69%, while it ranged between 53% and 67% in other European countries. Unfortunately, we were not able to show the difference between specific tumors types such as hormone receptor positive (HR+) or human epidermal growth factor 2 positive (HER2+) tumors as these specific data were not available. Nevertheless, it is known that the overall survival of women diagnosed with HR+/HER2+ is better when compared to triple negative breast cancers, since HR+/HER2+ tumors may respond to targeted therapies. Further research could investigate the impact of these specific tumor types on the burden of breast cancer such as incidence and survival. Although this would not change the overall burden of the disease, it will give us more understanding in inequality in burden between different types of breast cancer, also providing insights for further research and recourse allocation.

### Limitations

Although we were able to stratify most of the results by age and cancer stage, this was unfortunately not possible for the economic burden due to the unavailability of these data. Data were only available on total healthcare expenditures and could not stratified on expenditures by stage of diagnosis. Apart from availability of data, the changing health related burden over time is difficult to assess, as the TNM classification changed over time. The switch from the 5th to the 6th TNM classification, for example, resulted in a shift from stage IIa to stage I as well as a shift from stage IIb to stages III, which complicates the comparison of the burden over time. To overcome these, we grouped the stages by early, locally advanced and metastatic breast cancer, but also presented all data for the individual stages. Another limitation was related to the estimation of the proportion of health-care expenditure. Since 2009 oncology drugs are financed from the hospital budget instead of the general drug budget limiting the transparency of the actual drug costs due to breast cancer.

## Conclusion

This study provides a comprehensive assessment of the burden of breast cancer and subsequent trends over time in the Netherlands. Despite the promising trend of increase in survival after breast cancer diagnosis, this study shows that the BoD due to breast cancer remains significant. Furthermore, our study shows large differences in the incidence and mortality due to early, locally advanced, and metastatic breast cancer in the Netherlands.

### Additional file

Additional file 1: Productivity losses due breast cancer-related morbidity and mortality. (DOCX 51 kb)

## Figures and Tables

**Fig. 8:**
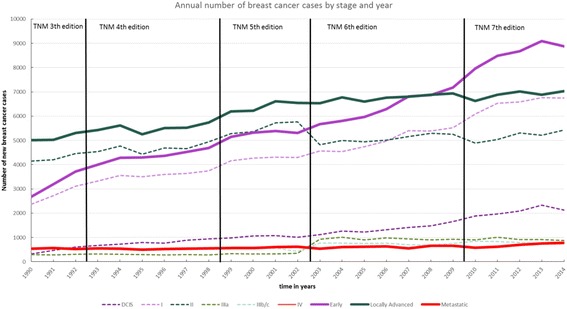
Total Incidence of breast cancer by stage between 1990 and 2014 for the Netherlands in absolute numbers. Vertical lines represents changes in TNM classification

## Abbreviations

BoD: Burden of Disease
CPI: Customer Price Index
DALYs: Disability Adjusted Life Years
DCIS: Ductal Carcinoma In Situ
ESR: European age Standardized Rates
HER2 +: Human Epidermal Growth factor 2 Positive
HR +: Hormone Receptor Positive
IARC: International Agency for Research on Cancer
ICD-O: International Classification of Diseases for Oncology
IKNL: Netherlands Comprehensive Cancer Organization
NCR: National Cancer Registry
RIVM: National Institute for Public Health
TNM: Tumour Node Metastasis
YLD: Years Lived with Disability
YLL: Years of Life Lost due to premature mortality

## References

[1] Jemal A (2011). Global cancer statistics. CA Cancer J Clin.

[2] Society AC (2012). Global Cancer Facts & Figures.

[3] Ferlay J (2013). Cancer incidence and mortality patterns in Europe: estimates for 40 countries in 2012. Eur J Cancer.

[4] IARC W (2012). GLOBOCAN 2012: estimated cancer incidence, mortality and prevalence worldwide in 2012.

[5] van der Waal D (2015). Breast cancer diagnosis and death in the Netherlands: a changing burden. Eur J Pub Health.

[6] Kruijshaar ME, Barendregt JJ (2004). The breast cancer related burden of morbidity and mortality in six European countries: the European disability weights project. Eur J Pub Health.

[7] Louwman WJ (2008). On the rising trends of incidence and prognosis for breast cancer patients diagnosed 1975-2004: a long-term population-based study in southeastern Netherlands. Cancer Causes Control.

[8] Fitzmaurice C (2015). The global burden of cancer 2013. JAMA Oncol.

[9] National Institute for Public Health and the Environment / Ministry of Health, Welfare and Sport. National Evaluation of breast cancer screening in the Netherlands (facts and figures). https://www.rivm.nl/en/Documents_and_publications/Common_and_Present/Publications/Disease_prevention_and_healthcare/breastcancer_screening/National_evaluation_of_breast_cancer_screening_in_the_Netherlands. Accessed 3 Oct 2014.

[10] Integraal Kankercentrum Nederland (IKNL), Dutch cancer registry. Cijfers over kanker. https://www.cijfersoverkanker.nl. Accessed apr 2017.

[11] James D. Brierley (Editor), Mary K. Gospodarowicz (Editor), Christian Wittekind (Editor). Union for International Cancer Control (UICC). The TNM Classification of Malignant Tumours, 8th Edition. ISBN: 978-1-119-26357-9. Jan 2017, Wiley-Blackwell.

[12] Cancer Research UK. Breast cancer stages types and grades. http://www.cancerresearchuk.org/about-cancer/breast-cancer/stages-types-grades/tnm-staging. Accessed Apr 2017.

[13] Soerjomataram I (2006). Increased risk of second malignancies after in situ breast carcinoma in a population-based registry. Br J Cancer.

[14] Knowlegde Institute of Medical Specialist. Richtlijnen database. Guidelines for the treatment of breast cancer in The Netherlands. https://richtlijnendatabase.nl/en/richtlijn/breast_cancer/breast_cancer.html. Accessed Apr 2017.

[15] Central Bureau for Statistics (CBS). Lifetable; life expectancy by gender and age in the Netherlands. http://statline.cbs.nl/Statweb/publication/?DM=SLNL&PA=37360ned&D1=0&D2=a&D3=a&D4=l&HDR=G1,T&STB=G2,G3&VW=T. Accessed Apr 2017.

[16] Minicozzi P, Otter R, Primic-Žakelj M, Francisci S. Survival of Cancer Patients in Europe, 1999–2007: The EUROCARE-5 Study. European Journal of Cancer. 2015;51(15):2099–2268. 10.1016/j.ejca.2015.08.019 26421814

[17] Stouthard MEA, Essink-Bot M-L, Bonsel G, Barendregt JJM, Kramers PGN, van de Water HPA, yvan der Maas PJ. Disability Weights for Diseases in the Netherlands. Rotterdam: Department of Public Health, Erasmus University; 1997. https://www.researchgate.net/publication/254787050_Disability_Weights_for_Diseases_in_The_Netherlands.

[18] Ministry of public health, wellbeing and sport (RIVM). Cost of illness database. https://kostenvanziektentool.volksgezondheidenzorg.info/tool/nederlands/?ref=kvz_v2l1b1p4r1c2i0t1j0o1y6a-1g0d14s54z0f0w2. Accessed Apr 2017.

[19] Central Bureau for Statistics (CBS). Customer Price Index (CPI) for The Netherlands. http://statline.cbs.nl/StatWeb/publication/?VW=T&DM=SLEN&PA=83131ENG&LA=EN. Accessed Apr 2017.

[20] Hanly P (2012). Breast and prostate cancer productivity costs: a comparison of the human capital approach and the friction cost approach. Value Health.

[21] Marmot MG (2013). The benefits and harms of breast cancer screening: an independent review. Br J Cancer.

[22] Bleyer A, Welch HG (2012). Effect of three decades of screening mammography on breast-cancer incidence. N Engl J Med.

[23] Lowy DR, Collins FS (2016). Aiming high--changing the trajectory for cancer. N Engl J Med.

[24] Hanahan D (2014). Rethinking the war on cancer. Lancet.

[25] Golubnitschaja O (2016). Breast cancer epidemic in the early twenty-first century: evaluation of risk factors, cumulative questionnaires and recommendations for preventive measures. Tumour Biol.

[26] Hofvind S (2012). Breast cancer incidence trends in Norway--explained by hormone therapy or mammographic screening?. Int J Cancer.

[27] Renard F, Van Eycken L, Arbyn M (2011). High burden of breast cancer in Belgium: recent trends in incidence (1999-2006) and historical trends in mortality (1954-2006). Arch Public Health.

[28] Pollán M (2009). Recent changes in breast cancer incidence in Spain, 1980–2004. J Natl Cancer Inst.

[29] Balasubramaniam SM, Rotti SB, Vivekanandam S (2013). Risk factors of female breast carcinoma: a case control study at Puducherry. Indian J Cancer.

[30] Oh H (2015). *The interaction between early-life body size and physical activity on risk of breast cancer.* International journal of cancer. Int J Cancer.

[31] Stoll BA (1998). Western diet, early puberty, and breast cancer risk. Breast Cancer Res Treat.

[32] Talma H (2013). Trends in menarcheal age between 1955 and 2009 in the Netherlands. PLoS One.

[33] van Gemert WA (2015). The proportion of postmenopausal breast cancer cases in the Netherlands attributable to lifestyle-related risk factors. Breast Cancer Res Treat.

[34] Walters S (2013). Breast cancer survival and stage at diagnosis in Australia, Canada, Denmark, Norway, Sweden and the UK, 2000-2007: a population-based study. Br J Cancer.

