# Supplementary material for: Burden of early, advanced and metastatic breast cancer in The Netherlands
Source: BMC Cancer. 2018 Mar 7;18:262. doi: 10.1186/s12885-018-4158-3 (PMC5842550; doi:10.1186/s12885-018-4158-3)
Supplement: Supplementary file 1 — Productivity losses due breast cancer-related morbidity and mortality. (DOCX 51 kb) [file 12885_2018_4158_MOESM1_ESM.docx]

**Additional file**

**Additional file 1 Productivity losses due breast cancer-related morbidity and mortality**

The Human Capital Approach was used to estimate the cost of mortality and morbidity related productivity losses due to breast cancer in The Netherlands. This approach measures the total value of potential productivity from a societal perspective. To estimate the productivity losses due to breast cancer-related deaths, first the number of breast cancer related deaths were obtained from the IKNL website [1]. Three measure were used to estimate the cost of productivity losses: Years of Potential Life years Lost (YPLL), Years of Potential productive life lost (YPPLL) and the cost of lost productivity (CLP) due to premature mortality and morbidity. To estimate YPLL and CPL we used the life expectancy of the general Dutch population retrieved from the Central bureau of statistics[2] and applied a working age from 15 to 65 years.

Estimation methods

YPLL Estimation Due to Premature Mortality: To estimate YPLL for premature mortality due to breast cancer, all the deaths in each age group where assumed to occur in the mid-point of that age group. The number of deaths in each age group were multiplied by the average of the remaining life expectancy for that age group to calculate the YPLL. The total number of YPLL was calculated using the following equation:

$$YPLL=\sum_{i=0}^{\infty} di\times Li$$

In this formula di represents the number of deaths at the mid-point of each age group (i) and Li is the average remaining life expectancy for that age group (i) would they not have died from breast cancer, based on the average life expectancy in the Netherlands.

To calculate the cost of productivity lost due to premature mortality we first estimated the Years of Potential Productive Life Lost (YPPLL) were calculated. The YPPLL estimation is similar to YPLL although deaths below age 15 and above age 65 where disregarded to calculate YPPLL and the number of life years lost where limited to the age or retirement (65 years for this study). The YPPLL was calculated by the following equation:

$$YPPLL=\sum_{i=0}^{N} di\times\left( W_{U}-W_{L} \right)$$

Here di is the number of death at the mid-point of each age group (i), W_U_ is the age of retirement (65), and W_L_ is the lowest productive age (15).

Third, the calculated YPPLL was multiplied by age and sex-specific annual wages (women) from age of deaths until the retirement age. The estimated costs of lost productivity were adjusted based on the employment and housekeeping rate for the year of interest. Moreover, to obtain the present value, the future costs were discounted at 4% per year. Future wages where adjusted for the average annual wage growth rate for the Netherlands which was 1.5% between 1991 and 2016. Cost of productivity loss for each death was summed across age groups.

Productivity losses due to morbidity where calculated the same way although the time frame was here limited from the moment of diagnosis until median duration of absence from work which was 17 months in the Netherlands. Additionally, a factor was taken into account for the fraction of patients that stop working after being diagnosed with breast cancer which was 75% of the breast cancer patients in the Netherlands. This Data on temporary disability as time absent from work for each patient was obtained from both Cancer for Care, an organization for re-integration of cancer patients, and data from the Boer et al [4].

Table S1. 10-year overall survival for breast cancer between 1989 and 1992 by stage of diagnosis

Table S2. 10-year overall survival for breast cancer between 1992 and 1998 by stage of diagnosis

Table S3. 10-year overall survival for breast cancer between 1999 and 2002 by stage of diagnosis

Table S4. 10-year overall survival for breast cancer between 2003 and 2009 by stage of diagnosis

Table S5. 10-year overall survival for breast cancer between 2010 and 2013 by stage of diagnosis

Table S6: Absolute number of Life-years lost due to early mortality by breast cancer

| Year | Total Life-years Lost (YLL) | YLL in good health |
| --- | --- | --- |
| 1990 | 54,261 | 31,887 |
| 1995 | 54,399 | 30,567 |
| 2000 | 54,121 | 29,069 |
| 2005 | 53,213 | 27,801 |
| 2010 | 51,931 | 28,100 |
| 2014 | 45,773 | 25,687 |
| Difference 1990-2014 | -15.6% | -19.4% |

**Additional file 1 Sensitivity analysis**

The sensitivity analysis showed that productivity losses due to morbidity were always between €258 million and €334.6 million per year. Productivity losses due to mortality ranged between €221 million and €323 million annually. The biggest impact on productivity losses due to morbidity were the emancipation rate and the income inequalities for male and female. For productivity losses due to mortality the discount rate and the productivity losses due to mortality had the highest impact.

Discounting and income growth rate is irrelevant using the Friction cost approach (FCA), since the FCA only takes 85 calendar days into account for productivity losses. To take emancipation into account female gross labor participation rates were changed to equal those of men, which was 40.4% for age group 15-25, 91.8% for 25-45 and 82.4% for 45-65.

Table S7: Sensitivity Analysis for productivity losses

|  | Productivity Losses due to morbidity (€) | Productivity losses due to mortality (€) |
| --- | --- | --- |
| **Base Case** | **259,778,871** | **242,713,394** |
| GDP growth 0% | 258,669,021 | 220,980,308 |
| GDP growth 3.5% | 261,258,671 | 277,762,688 |
| Discount rate 0% | 262,782,865 | 323,328,030 |
| Discount rate 2% | 261,251,417 | 277,571,002 |
| Discount rate 6% | 258,361,893 | 215,540,467 |
| Emancipation | 334,588,716 | 317,710,742 |
| Friction Cost Approach | 43,007,445 | 6,098,828 |

**Additional file 1. DALY Calculation**

One Disability Adjusted Life Year (DALY) represents one lost year of life in perfect health. The total number of DALYs lost due to a disease can be seen as measurement of the gap between the current status and the ideal health situation where all patients would live to an advanced age, free of disability [5].

The total DALYs are calculated by using both annual incidence and mortality data from 1990 until 2014 using the following equation:

$$DALY=YLL+YLD$$

Where YLL is the sum of years of life lost by patients with breast cancer due to premature mortality in a certain year of interest. The number of life years lost due to early mortality depends on the standard life expectancy at the age of death in years. The YLL is calculated by the following equation:

$$YLL=N\times L$$

where N = the number of deaths in the corresponding year and L= the standard life expectancy at the age of death in years.

The YLD is the number of years lost due to disability by living with breast cancer. To estimate YLD for breast cancer in a particular year, the number of incident cases in that year are multiplied by the average duration of the disease and the weight factor that reflects the severity of breast cancer on a scale from 0 (perfect health) to 1 (dead).

$$YLD=I\times DW\times L$$

where I = the number of incident cases in the year of interest, DW = the disability weight corresponding with breast cancer, which is 0.38 on average for in The Netherlands [6] and L = the average duration of breast cancer from the moment of diagnosis until remission or death which is 4.3 years in the Netherlands [7]. Life years lost were discounted by a discount rate of 1.5% which is the standard rate for utilities in The Netherlands.

1. (IKNL), D.C.R.N.C.C.C.N., *Cijfers over kanker.* 2016.

2. (CBS), D.C.B.f.s., *Life expectancy of women in the Netherlands.* 2016.

3. (CBS), D.C.B.f.S., *Consumer Price Index for the Netherlands (CPI).* 2017.

4. de Boer, A.G.E.M., et al., *Work ability and return-to-work in cancer patients.* British Journal of Cancer, 2008. **98**(8): p. 1342-1347.

5. Worl Health Organization (WHO), Health statistics ad information systems; Metrics: Disability-Adjusted Life Year (DALY). retrieved from: http://www.who.int/healthinfo/global_burden_disease/metrics_daly/en/ assesed 13 nov 2017

6. Stouthard MEA, Essink-Bot ML, Bonsel GJ, et al: Disability Weights for Diseases in The Netherlands. 1997, Rotterdam: Department of Public Health, Erasmus University of Rotterdam

7. EUROCARE-4 study
